# Development and validation of an algorithm using health administrative data to define patient attachment to primary care providers

**DOI:** 10.1108/JHOM-05-2020-0171

**Published:** 2021-07-26

**Authors:** Liisa Jaakkimainen, Imaan Bayoumi, Richard H. Glazier, Kamila Premji, Tara Kiran, Shahriar Khan, Eliot Frymire, Michael E. Green

**Affiliations:** Primary Care and Health Systems, ICES , Toronto, Canada; Department of Family and Community Medicine and the Institute of Health Policy, Management and Evaluation, University of Toronto , Toronto, Canada; Department of Family and Community Medicine, Sunnybrook Health Sciences Centre , Toronto, Canada; Department of Familty Medicine , Queens University, Kingston, Canada; ICES , Toronto, Canada; Department of Family and Community Medicine, St. Michael’s Hospital , Toronto, Canada; Department of Family and Community Medicine, University of Toronto , Toronto, Canada; MAP Centre for Urban Health Solutions, St. Michael’s Hospital , Toronto, Canada; Central Ottawa Family Medicine Associates , Ottawa, Canada; Department of Family Medicine, University of Ottawa , Ottawa, Canada; Department of Family Medicine, Western University , London, Canada; Department of Family and Community Medicine, St. Michaels’s Hospital, University of Toronto , Toronto, Canada; MAP Centre for Urban Health Solutions, Li Ka Shing Knowledge Institute, St. Michael’s Hospital , Toronto, Canada; Institute of Health Policy, Management and Evaluation, University of Toronto , Toronto, Canada; ICES , Toronto, Canada; ICES, Queens University , Kingston, Canada; Health Services and Policy Research Institute, Queen’s University , Kingston, Canada; Departments of Family Medicine, Health Services and Policy Research Institute , Kingston, Canada

**Keywords:** Primary care, Health services research, Methodology, Big data

## Abstract

**Purpose:**

The authors developed and validated an algorithm using health administrative data to identify patients who are attached or uncertainly attached to a primary care provider (PCP) using patient responses to a survey conducted in Ontario, Canada.

**Design/methodology/approach:**

The authors conducted a validation study using as a reference standard respondents to a community-based survey who indicated they did or did not have a PCP. The authors developed and tested health administrative algorithms against this reference standard. The authors calculated the sensitivity, specificity positive predictive value (PPV) and negative predictive value (NPV) on the final patient attachment algorithm. The authors then applied the attachment algorithm to the 2017 Ontario population.

**Findings:**

The patient attachment algorithm had an excellent sensitivity (90.5%) and PPV (96.8%), though modest specificity (46.1%) and a low NPV (21.3%). This means that the algorithm assigned survey respondents as being attached to a PCP and when in fact they said they had a PCP, yet a significant proportion of those found to be uncertainly attached had indicated they did have a PCP. In 2017, most people in Ontario, Canada (85.4%) were attached to a PCP but 14.6% were uncertainly attached.

**Research limitations/implications:**

Administrative data for nurse practitioner's encounters and other interprofessional care providers are not currently available. The authors also cannot separately identify primary care visits conducted in walk in clinics using our health administrative data. Finally, the definition of hospital-based healthcare use did not include outpatient specialty care.

**Practical implications:**

Uncertain attachment to a primary health care provider is a recurrent problem that results in inequitable access in health services delivery. Providing annual reports on uncertainly attached patients can help evaluate primary care system changes developed to improve access. This algorithm can be used by health care planners and policy makers to examine the geographic variability and time trends of the uncertainly attached population to inform the development of programs to improve primary care access.

**Social implications:**

As primary care is an essential component of a person's medical home, identifying regions or high need populations that have higher levels of uncertainly attached patients will help target programs to support their primary care access and needs. Furthermore, this approach will be useful in future research to determine the health impacts of uncertain attachment to primary care, especially in view of a growing body of the literature highlighting the importance of primary care continuity.

**Originality/value:**

This patient attachment algorithm is the first to use existing health administrative data validated with responses from a patient survey. Using patient surveys alone to assess attachment levels is expensive and time consuming to complete. They can also be subject to poor response rates and recall bias. Utilizing existing health administrative data provides more accurate, timely estimates of patient attachment for everyone in the population.

## Introduction

Access to primary care is the foundation of a high functioning health care system ( Starfield *et al.* , 2005 ). Lack of attachment to primary care is associated with multiple patient level and health system level problems. Unattached patients experience lower quality of care, higher inpatient hospitalization and readmission rates and higher ER utilization ( Olsen *et al.* , 2017 ; Shea *et al.* , 1992a , b ; Shi *et al.* , 1999 ; Ramondetta *et al.* , 2015 ; Ohle *et al.* , 2017 ; Farion *et al.* , 2015 ; Estrada and Ownby, 2017 ). Having a regular primary care provider (PCP) has also been linked with better patient experiences including greater trust and confidence in care and more personalized care ( von Bultzingslowen *et al.* , 2006 ). In Canada, many provinces have identified the critical need for attachment to a PCP and a variety of approaches to address have been implemented ( Breton *et al.* , 2017 ). It is critical for health system planners to efficiently assess numbers, locations and profiles of unattached patients to enable data-guided health human resource and program planning.

Multiple researchers have identified valid approaches for identifying physician patient profiles in administrative data, ( Lasko *et al.* , 2006 ; Katz *et al.* , 2004 ) but these methods do not necessarily identify unattached patients. Provost *et al.* (2015) developed an algorithm to identify unattached patients in administrative data, but were unable to validate their approach with patient survey data. We sought to develop and validate an algorithm using health administrative data to identify patients who are attached to consistent PCP using patient responses to the Health Care Experiences Survey (HCES) in Ontario, Canada.

## Objectives

The objectives for this study were to develop and validate a patient attachment algorithm for persons over 16 years of age using health administrative data and to apply this algorithm to the entire population of Ontario, Canada.

## Methods

Study method: We conducted a validation study of respondents to the HCES conducted by the Ontario Ministry of Health (n.d.) . The HCES is a voluntary, telephone survey conducted quarterly. We used survey data between October 2012 to September 2018 and included Ontario residents aged 16 years of age and older. The sampling frame was weighted to geographic regions in Ontario called Local Health Integration Networks (LHINs) and over-sampled rural areas.

Reference standard: Our reference standard included the HCES respondents who indicated they did or did not have a PCP. We compared the responses from HCES respondents to their actual health care use of PCPs by examining Ontario health administrative data. For this study, primary care use meant being enrolled with a family physicians' (FPs’) practice (rostered) or visiting a FP.

Study cohort: The study cohort included respondents to the HCES who consented to have their questionnaires linked to the Ontario health administrative data held at ICES. ICES is an independent, non-profit research institute whose legal status under Ontario's health information privacy law allows it to collect and analyze health care and demographic data, without consent, for health system evaluation and improvement ( ICES, n.d. ). The vast majority (92%) of the HCES respondents were linked to the Ontario health administrative data ( Ontario Ministry of Health, n.d. ).

Health administrative data: Several sources of health administrative data were used to identify primary care use. In Ontario, and elsewhere in Canada, FPs are the physician providers of primary care for adults and most children ( Jaakkimainen *et al.* , 2006 ). The Client Agency Program Enrollment (CAPE) database was used to identify patients who were enrolled with a FP belonging to a primary care patient enrollment model (PEM) in Ontario ( McLeod *et al.* , 2016 ). There are several types of PEMs in Ontario which formally enroll (roster) patients to a FP, including those remunerated through blended capitation (age- and sex-adjusted monthly payments for each enrolled patient plus a small proportion of fee-for-service payments) and those primarily paid by fee-for-service. A community health centre (CHC) database identified patients visiting a CHC, where FPs are salaried and funded under a global budget. Finally, the Ontario Health Insurance Plan (OHIP) database contains fee-for-service physician claims for all physicians in Ontario. For this study, primary care core visits refer to a list of services determined to be part of a comprehensive primary care practice ( Schultz and Glazier, 2017 ). Hospital-based healthcare use referred to any emergency department (ED) visit or hospitalizations. ED visits came from the Canadian Institute for Health Information (CIHI) National Ambulatory Record System (NACRS) database and hospital admissions came from the CIHI Discharge Abstract Database (DAD). We included all acuity (Canadian Triage and Acuity Scale (CTAS)) levels of ED visits ( Fernandes *et al.* , 2013 ). These datasets were linked using unique encoded identifiers and analyzed at ICES.

Patient characteristics: Patient age and sex were determined from the Ontario provincial health registry called the Registered Persons Database (RPDB). Neighborhood income quintile was derived by linking postal code to census dissemination area ( Statistics Canada, 2013 ). Rurality was determined by linking postal code with the Rurality Index of Ontario ( Kralj, 2000 ).

Patient attachment algorithm using health administrative data: We developed hierarchical steps in attributing a HCES respondent as being attached to a PCP with the order being set by those steps that attached the largest numbers of patients. Under the Canada Health Act, all residents in each province are entitled to publicly funded healthcare services ( Marchildona and Hutchison, 2016 ). Consequently, all provinces in Canada collect physicians’ claims data. Primary care reform in Ontario started in 2002 and saw a large increase in FPs participating in formal primary care patient enrollment (roster) models (PEMs) ( Marilisa Tiedemann, 2020 ). However, formal primary care PEMs are not available in all Canadian provinces. We developed a patient attachment algorithm which includes steps using PEMs if they are available in a jurisdiction. However, if not available, then physician claims data can be used.

First, HCES respondents found to be enrolled in a PEM were considered as having a PCP. Enrollment is the system requirement in establishing a connection to a PCP and is the health system indicator of attachment. Second, HCES respondents seen at a CHC were considered as having a PCP. Third, we defined HCES respondents as being “virtually” enrolled to a PCP with whom the plurality of their primary care core visits were made over a two-year period. All OHIP PCP claims by HCES respondents were extracted with only one claim per patient per PCP physician per day being counted. Total primary care core visits to each individual PCP and total primary care core visits per patient were counted. However, we did not want to virtually enroll a HCES respondent to a PCP whom themselves may have low continuity of care (CoC) with their patients, such as walk-in clinic PCPs. Therefore, we calculated a PCP CoC index which is a visit-based measure of the proportion of an individual PCP visits over all physician's visits seen over a two-year time period ( Jee and Cabana, 2006 ). The PCP CoC index was determined with a numerator of patients virtually rostered to a PCP divided by the denominator of all unique patients the same PCP had seen over two years. If the PCP CoC was less than or equal to 10%, then this PCP had a low PCP CoC and HCES respondents virtually enrolled to these PCPs were then deemed to be uncertainly attached. Therefore, patients were deemed attached to a PCP if they belonged to a PEM, were seen in a CHC or they were virtually enrolled to a PCP who had a PCP CoC index over 10%. Otherwise, patients were deemed uncertainly attached. We used the term uncertainly attached as opposed to unattached because patients could and in fact did still access PCPs (for example episodic care in walk in clinics), even when they said they did not have access to ongoing primary care.

Validation analyses: We conducted several analyses to assess the impact of assumptions made in developing the patient attachment algorithm. We split the study cohort into a development dataset and a validation dataset. The development dataset consisted of all HCES respondents linked with ICES data and surveyed between October 2012 and September 2017 ( *N* = 39,285) and the validation dataset consisted of HCES respondents linked with ICES surveyed between October 2017 and September 2018 ( *N* = 6,621). The HCES responses to the question of whether they had or did not have a PCP were compared to each step of the patient attachment algorithm (described above). In addition, the third step of the patient attachment algorithm whereby HCES respondents were virtually enrolled to a PCP were examined by comparing a cut-point for PCP CoC index of less than 25%. Finally, we examined whether the remaining uncertainly attached patients had any primary care core visits or any hospital-based healthcare use in the two years prior to their completing the HCES survey.

Statistical analysis: The sensitivity, specificity, positive predictive value (PPV) and negative predictive value (NPV) were calculated for the overall patient attachment algorithm. For the validation analyses two-sample *t* -tests were undertaken to compare proportions between groups, with *p* < 0.001 indicating statistical significance. All analyses were performed using SAS Enterprise Guide version 7.1 (Cary, NC) ( SAS, n.d. ).

Application of the Patient Attachment Algorithm to the Ontario population: The patient attachment algorithm was applied to the 2017 population of Ontario. The 2017 Ontario population included all residents with a valid health care number and who were alive as of December 31, 2017. As the HCES survey is conducted for resident ages 16 years and older, our patient attachment algorithm was developed for people over 16 years of age. However, for the application of our algorithm to the entire Ontario population in 2017, we added another step where we applied a health administrative data pediatric access algorithm for children under 19 years of age. This algorithm has been previously validated for pediatric health services research to examine primary care access in pediatric populations ( Guttmann *et al.* , 2010 ). If children under 19 years of age were not attached to a PCP after applying our three patient attachment algorithm steps (which were validated against HCES respondents aged 16 years and older), we then assigned them to a PCP based on the pediatric access algorithm.

The use of data in this project was authorized under section 45 of Ontario's Personal Health Information Protection Act, which does not require review by a Research Ethics Board.

## Results

Overall, 55,392 HCES respondents were linked to the Ontario health administrative data between 2012 and 2018, of which 52,504 (94.8%) indicated they had a PCP and 2,888 (5.2%) indicated they did not. Table 1 presents the demographic characteristics of the HCES respondents. A significantly higher proportion of HCES respondents attached to PCP were older, female, lived in urban areas and in higher income neighborhoods than HCES respondents uncertainly attached to PCP.

**Figure 1:**
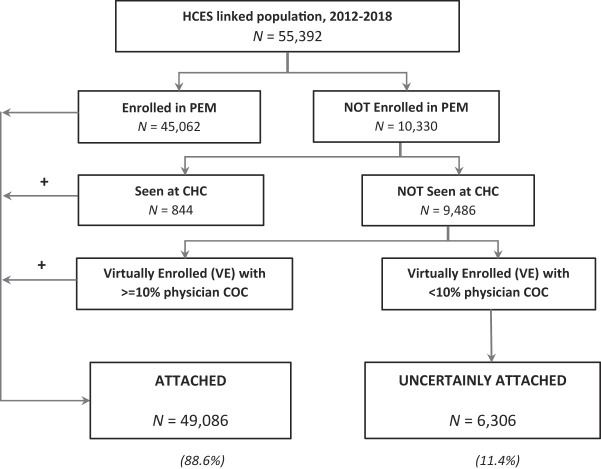
Flowchart of the steps for the patient attachment algorithm validation

A flowchart of the HCES respondent attachment algorithm using health administrative data is provided in Figure 1 . 81.4% of HCES respondents were rostered to a PEM and 1.5% were seen in a CHC. Of the 9486 HCES respondents not rostered to a PEM or seen in a CHC, 3,180 (33.5%) were virtually rostered to a PCP with greater than or equal to 10% FP CoC index. Overall, 88.6% of HCES respondents were defined as being attached to a PCP or group using health administrative data. The remaining 6,306 (11.4%) of HCES respondents were deemed uncertainly attached.

The validation analyses found no statistically significant differences between the development dataset and the validation dataset in any step of the patient attachment algorithm. Using a PCP CoC index cut point of less than 10% had a higher proportion of HCES respondents reporting they had no PCP and being attributed to the uncertainly attached group (true negative) when compared to a less than 25% cut point. There was no difference in the proportion of HCES respondents attributed to the attached group using either PCP CoC index cut point of less than 10% or 25% and reporting they had a PCP (true positive).

Amongst the 6306 HCES patients who remained uncertainly attached using the algorithm, 4,416 (70%) had at least one core primary care visits in the two years prior to their completing the HCES survey. For the subgroup of HCES respondents uncertainly attached and saying they did not have a PCP, we found 225/692 (32.5%) did use health care services (hospital-based healthcare use) in the two year prior to their completing the survey. For HCES respondents with a child less than 16 years of age, 94.2% indicated their child had a FP or pediatrician.

The patient attachment algorithm for adults over 16 years of age had a sensitivity of 90.5%, specificity of 46.1%, a PPV of 96.8% and a NPV of 21.3% (see Table 2 ). In other words, our patient attachment algorithm identified 90.5% of HCES respondents who said they had a PCP, as being attached to a PCP. However, our algorithm identified 46.1% of HCES respondents who said they did not have a PCP as being uncertainly attached to a PCP.

**Figure 2:**
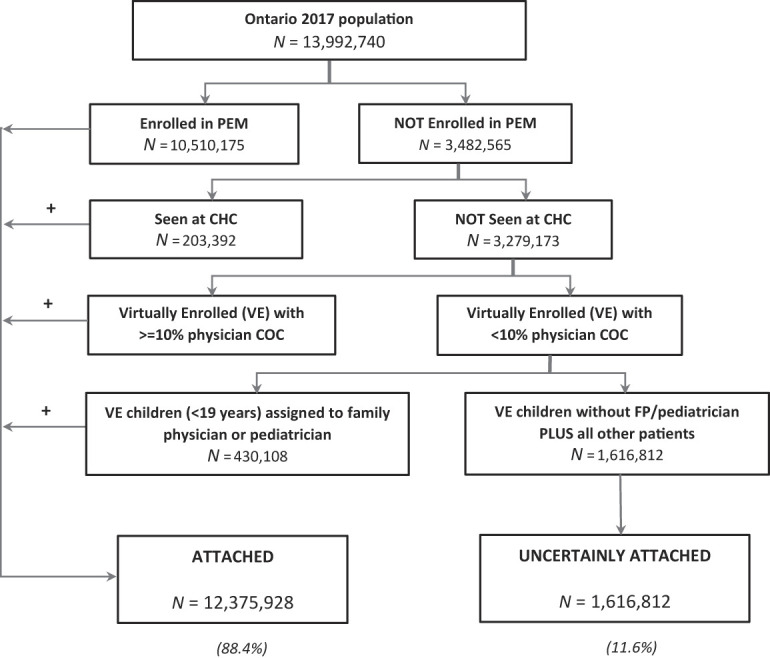
Flowchart of the patient attachment algorithm applied on the ontario 2017 population

The patient attachment algorithms and the pediatric access algorithms were applied to the 2017 Ontario population ( Figure 2 ), 88.4% of the Ontario population were attached, and 11.6% were uncertainly attached to a PCP.

## Discussion

In 2017, most people in Ontario, Canada (88.4%) were attached to a PCP but 11.6% were uncertainly attached. The patient attachment algorithm had an excellent true positive rate (sensitivity and PPV) meaning the algorithm identified HCES respondents as being attached to a PCP when HCES respondents themselves indicated they have a PCP. But our algorithm had a modest specificity (true negative rate) and a low NPV. This means that while the algorithm identified HCES respondents as being uncertainly attached does not necessarily indicate that they did not have access to a PCP. Some people may feel healthy and may not feel the need to seek medical care. Others may visit walk-in clinics on a needed basis and may not feel they need to see the same PCP. Indeed we found one-third of HCES respondents who were uncertainly attached to a PCP and indicated they did not have a PCP, did use health care services in the two years prior to their completing the survey.

Our study was able to link the responses from individuals about their primary care attachment with their actual use of the primary care system. Previous work conducted in Ontario in 2007 and 2008 had used a patient survey alone to estimate 92.9% (95% CI: 92.4, 93.4) of people over 16 years of age were attached to a PCP ( Hay *et al.* , 2010 ). The HCES similarly found 94.8% of respondents saying they had a PCP. Patient surveys are expensive and time consuming to complete. They can also be subject to sampling bias including poor response rates, recall bias and not capturing those without a phone. Utilizing existing health administrative data can provide more accurate, timely estimates of patient attachment for everyone in the population.

In Canada, medically necessary physician visits are universally provided to residents and paid and managed by provincial government health plans ( Government of Canada, n.d. ). The Ontario primary care system includes both formal (patient rostered) and informal enrolment models of care. Our attachment algorithm incorporates both types of enrolment methods. For jurisdictions that do not have a formal enrolment system or rostering of patients to PCP, they could still use our algorithm by applying our “virtual enrollment” method of attaching patients based on their visit patterns to a PCP. Similarly, primary care systems that are mainly based on formal enrolment methods can also use our algorithm. In Ontario patients are enrolled (rostered) to an individual PCP and not to a practice. Most PCPs that enroll patients, practice in a group primary care practice setting with other PCPs who enroll patients ( Glazier *et al.* , 2012 ). PCPs may work in other clinical settings such as EDs or nursing homes. In our algorithm, patients seen in these settings would not be attributed to the PCP as these encounter claim codes are specific to EDs or nursing home locations. Our algorithm will attach patients to individual PCPs, though the reality is most PCPs work in a group setting.

Information about people uncertainly attached to a PCP is needed by health care planners, decisions makers and policy makers. As primary care is an essential component of a person's medical home, identifying regions or high need populations that have higher levels of uncertainly attached patients will help target programs to support their primary care access and needs. Providing annual reports on uncertainly attached patients can also help evaluate primary care system changes developed to improve access. Furthermore, this approach will be useful in future research to determine the health impacts of uncertain attachment to primary care, especially in view of a growing body of literature highlighting the importance of primary care continuity.

There are limitations to our study. We only looked at FPs as the providers of primary care and we did not include nurse practitioners. For those patients in primary care teams we also did not look at care provided by interprofessional healthcare providers (e.g. pharmacists, social work). Nurse practitioner-led clinics (NPLC) are more common in rural and underserviced communities and in other provinces and there are currently 27 NPLCs in Ontario ( Nurse Practitioner Association of Ontario, n.d. ; College of Nurse of Ontar, 2017 ). Unfortunately, administrative data for nurse practitioner's encounters and other interprofessional care providers are not currently available. Health administrative data should strive to include encounter data from all primary health care providers as this can help monitor and evaluate the full picture of primary healthcare delivery. We also cannot separately identify primary care visits conducted in walk in clinics using our health administrative data. In addition, our definition of hospital-based healthcare use did not include outpatient specialty care. And finally, the HCES respondent sample is not generalizable to the entire Ontario population. The survey did oversample people living in rural communities and people who do not have a phone or are not able to provide answers over the phone were not included.

## Conclusions

We developed a patient attachment algorithm using existing health administrative data compared to responses from a population-based patient survey. This algorithm had an excellent sensitivity and PPV, though a modest specificity. It can be used by health care planners and policy makers to examine the geographic variability and time trends of the uncertainly attached population to inform the development of programs to improve primary care access.

## Figures and Tables

**Table 1:** Demographic characteristics of the attached and uncertainly attached HCES respondents

	Attached	Uncertainly attached	Total	Ontario population ≥ 16 years of age
Age (Mean ± SD)	52.99 ± 17.29	49.83 ± 17.54	52.63 ± 17.35	47.55 ± 18.84
*Age Groups*				
16–18	1,079 (2.2%)	202 (3.2%)	1,281 (2.3%)	475,431 (4.1%)
19–34	7,109 (14.5%)*	1,222 (19.4%)	8,331 (15.0%)	2,953,638 (25.4%)
35–49	12,194 (24.8%)	1,618 (25.7%)	13,812 (24.9%)	2,826,277 (24.3%)
50–64	14,842 (30.2%)	1,880 (29.8%)	16,722 (30.2%)	2,996,962 (25.8%)
65–74	8,422 (17.2%)*	851 (13.5%)	9,273 (16.7%)	1,329,508 (11.5%)
75–84	4,317 (8.8%)*	422 (6.7%)	4,739 (8.6%)	710,156 (6.1%)
85+	1,123 (2.3%)*	111 (1.8%)	1,234 (2.2%)	318,551 (2.7%)
*Sex*				
Female	28,469 (58.0%)*	3,143 (49.8%)	31,612 (57.1%)	5,971,110 (51.4%)
Male	20,617 (42.0%)*	3,163 (50.2%)	23,780 (42.9%)	5,639,413 (48.6%)
*Rurality*				
Urban	29,947 (61.0%)*	3,087 (49.0%)	33,034 (59.6%)	8,451,253 (72.8%)
Semi-urban	12,575 (25.6%)*	2,064 (32.7%)	14,639 (26.4%)	2,224,266 (19.2%)
Rural	5,859 (11.9%)*	843 (13.4%)	6,702 (12.1%)	839,468 (7.2%)
Missing	705 (1.4%)*	312 (4.9%)	1,017 (1.8%)	95,536 (0.8%)
*Income Quintiles*				
1 Low	8,030 (16.4%)*	1,293 (20.5%)	9,323 (16.8%)	2,112,944 (18.2%)
2	9,245 (18.8%)	1,213 (19.2%)	10,458 (18.9%)	2,237,908 (19.3%)
3	9,732 (19.8%)	1,216 (19.3%)	10,948 (19.8%)	2,320,365 (20.0%)
4	10,873 (22.2%)*	1,265 (20.1%)	12,138 (21.9%)	2,509,732 (21.6%)
5 High	11,021 (22.5%)*	1,275 (20.2%)	12,296 (22.2%)	2,380,346 (20.5%)
Missing	185 (0.4%)	44 (0.7%)	229 (0.4%)	49,228 (0.47%)

Note(s): * p < 0.001

**Table 2:** 2 × 2 Table of the Patient Attachment Algorithm again HCES respondents ( *N* = 55,392)

	HCES respondents indicating they did a primary care provider	HCES respondents indicating they did not have a primary care provider	
Algorithm Identified as being ATTACHED to a primary care provider	47,516	1,570	49,086
Algorithm Identified as being UNCERTAINLY ATTACHED to a primary care provider	4,963	1,343	6,306
	52,479	2,913	55,392

## References

[ref001] Breton , M. , Green , M. , Kreindler , S. , Sutherland , J. , Jbilou , J. , Wong , S.T. , Shaw , J. , Crooks , V.A. , Contandriopoulos , D. , Smithman , M.A. and Brousselle , A.A. ( 2017 ), “ A comparative analysis of centralized waiting lists for patients without a primary care provider implemented in six Canadian provinces: study protocol ”, BMC Health Services Research , Vol. 17 , p. 6 , doi: 10.1186/s12913-017-2007-8 . 28109279 PMC5251310

[ref002] College of Nurse of Ontario ( 2017 ), “ Membership statistics report 2017 pub. No. 43069 ISSN 1916 - 047X copyright © college of nurses of Ontario ”, available at: http://www.cno.org/globalassets/docs/general/43069_stats/2017-membership-statistics-report.pdf .

[ref003] Estrada , R.D. and Ownby , D.R. ( 2017 ), “ Rural asthma: current understanding of prevalence, patterns, and interventions for children and adolescents ”, Current Allergy and Asthma Reports , Vol. 17 No. 6 , p. 37 . 28484946 10.1007/s11882-017-0704-3 PMC6533905

[ref004] Farion , K.J. , Wright , M. , Zemek , R. , Neto , G. , Karwowska , A. , Tse , S. , Reid , S. , Jabbour , M. , Poirier , S. , Moreau , K.A. and Barrowman , N. ( 2015 ), “ Understanding low-acuity visits to the pediatric emergency department ”, Plos One [Electronic Resource] , Vol. 10 No. 6 , p. e0128927 . 10.1371/journal.pone.0128927 PMC4471269 26083338

[ref005] Fernandes , C.M. , McLeod , S. , Krause , J. , Shah , A. , Jewell , J. , Smith , B. and Rollins , L. ( 2013 ), “ Reliability of the Canadian Triage and Acuity Scale: interrater and intrarater agreement from a community and an academic emergency department ”, CJEM , Vol. 15 No. 4 , pp. 227 - 232 , doi: 10.2310/8000.2013.130943 . 23777994

[ref006] Glazier , R.H. , Zagorski , B.M. and Rayner , J. ( 2012 ), Comparison of Primary Care Models in Ontario by Demographics, Case Mix and Emergency Department Use, 2008/09 to 2009/10 ICES Investigative Report , Institute for Clinical Evaluative Sciences , Toronto .

[ref007] Government of Canada (n.d.), “ Canada's health care system ”, available at: https://www.canada.ca/en/health-canada/services/health-care-system/reports-publications/health-care-system/canada.html .

[ref008] Guttmann , A. , Shipman , S.A. , Lam , K. , Goodman , D.C. and Stukel , T.A. ( 2010 ), “ Primary care physician supply and children's health care use, access, and outcomes: findings from Canada ”, Pediatrics , Vol. 125 No. 6 , pp. 1119 - 1126 . 20498170 10.1542/peds.2009-2821

[ref009] Hay , C. , Pacey , M. , Bains , N. and Ardal , S. ( 2010 ), “ Understanding the unattached population in Ontario: evidence from the primary care access survey (PCAS) ”, Healthcare Policy = Politiques de sante , Vol. 6 No. 2 , pp. 33 - 47 . 22043222 PMC3016634

[ref010] ICES , *Privacy at ICES* (n.d.), Available at: https://www.ices.on.ca/Data-and-Privacy/Privacy-at-ICES ( accessed 20 April 2020 ).

[ref011] Jaakkimainen , L. , Schultz , S.E. , Klein-Geltink , J.E. , Thiruchelvam , D. and Kopp , A. ( 2006 ), “ Ambulatory physician care for adults and guttman A, schultz SE, Jaakkimainen L. Primary care for children ”, in Jaakkimainen , L. , Upshur , R. , Klein-Geltink , J.E. , Leong , A. , Maaten , S. , Schultz , S.E. and Wang , L. (Eds), Primary Care in Ontario: ICES Atlas , Institute for Clinical Evaluative Sciences , Toronto .

[ref012] Jee , S.H. and Cabana , M.D. ( 2006 ), “ Indices for continuity of care: a systematic review of the literature ”, Medical Care Research and Review , Vol. 63 No. 2 , pp. 158 - 188 , doi: 10.1177/1077558705285294 . 16595410

[ref013] Katz , A. , De Coster , C. , Bogdanovic , B. , Soodeen , R.A. and Chateau , D. ( 2004 ), Using Administrative Data to Develop Indicators of Quality in Family Practice , Manitoba Centre for Health Policy, University of Manitoba , Winnipeg .

[ref014] Kralj , B. ( 2000 ), “ Measuring ‘rurality’ for purposes of health care planning: an empirical measure for Ontario ”, Ontario Medical Review , Vol. 67 , pp. 33 - 5219 .

[ref015] Lasko , T.A. , Atlas , S.J. , Barry , M.J. and Chueh , H.C. ( 2006 ), “ Automated identification of a physician's primary patients ”, Journal of the American Medical Informatics Association , Vol. 13 No. 1 , pp. 74 - 79 . 16221940 10.1197/jamia.M1876 PMC1380200

[ref016] Marchildona , G.P. and Hutchison , B. ( 2016 ), “ Primary care in Ontario, Canada: new proposals after 15 years of reform ”, Health Policy , Vol. 120 , pp. 732 - 738 . 27160481 10.1016/j.healthpol.2016.04.010

[ref017] Marilisa Tiedemann ( 2020 ), Legal and Social Affairs Division. Parliament and Information Research Services. The Canada Health Act-An Overview (Background Paper) , Publication No. 2019-54-E , Library of Parliament , Ottawa , ( accessed 17 December 2019 ).

[ref018] McLeod , L. , Buckley , G. and Sweetman , A. ( 2016 ), “ Ontario primary care models: a descriptive study ”, CMAJ Open , November , Vol. 114 No. 4 , pp. E679 - E688 , doi: 10.9778/cmajo.20160069 . PMC5173461 28018882

[ref019] Nurse Practitioner Association of Ontario (n.d.), Available at: https://npao.org/about-npao/clinics/ .

[ref020] Ohle , R. , Ohle , M. and Perry , J.J. ( 2017 ), “ Factors associated with choosing the emergency department as the primary access point to health care: a Canadian population cross-sectional study ”, CJEM , Can. , Vol. 19 No. 4 , pp. 271 - 276 . 27514693 10.1017/cem.2016.350

[ref021] Olsen , C.G. , Boltri , J.M. , Amerine , J. and Clasen , M.E. ( 2017 ), “ Lacking a primary care physician is associated with increased suffering in patients with severe mental illness ”, Journal of Primary Prevention , Vol. 38 No. 6 , pp. 583 - 596 . 10.1007/s10935-017-0490-7 28929367

[ref022] Ontario Ministry of Health (n.d.), “ Ministry of long-term care ”, The Health Care Experience Survey , available at: http://www.health.gov.on.ca/en/common/healthcareexperiencesurvey.aspx ( accessed 20 April 2020 ).

[ref023] Provost , S. , Perez , J. , Pineault , R. , Borges Da Silva , R. and Tousignant , P. ( 2015 ), “ An algorithm using administrative data to identify patient Attachment to a family physician ”, International Journal of Family Medicine , Vol. 2015 , p. 11 , doi: 10.1155/2015/967230 . PMC4564640 26413320

[ref024] Ramondetta , L.M. , Meyer , L.A. , Schmeler , K.M. , Daheri , M.E. , Gallegos , J. , Scheurer , M. , Montealegre , J.R. , Milbourne , A. , Anderson , M.L. and Sun , C.C. ( 2015 ), “ Avoidable tragedies: disparities in healthcare access among medically underserved women diagnosed with cervical cancer ”, Gynecologic Oncology , Vol. 139 No. 3 , pp. 500 - 505 . 26498912 10.1016/j.ygyno.2015.10.017 PMC7418500

[ref025] SAS (n.d.), SAS Enterprise Guide Version 7.1 , SAS Institute, Cary, NC .

[ref026] Schultz , S.E. and Glazier , R.H. ( 2017 ), “ Identification of physicians providing comprehensive primary care in Ontario: a retrospective analysis using linked administrative data ”, CMAJ Open , December , Vol. 195 No. 4 , pp. E856 - E863 , doi: 10.9778/cmajo.20170083 . PMC5741421 29259018

[ref027] Shea , S. , Misra , D. , Ehrlich , M.H. , Field , L. and Francis , C.K. ( 1992 ), “ Predisposing factors for severe, uncontrolled hypertension in an inner-city minority population ”, New England Journal of Medicine , Vol. 327 No. 11 , pp. 776 - 781 . 10.1056/NEJM199209103271107 1501654

[ref028] Shea , S. , Misra , D. , Ehrlich , M.H. , Field , L. and Francis , C.K. ( 1992 ), “ Correlates of nonadherence to hypertension treatment in an inner-city minority population ”, American Journal of Public Health , Vol. 82 No. 12 , pp. 1607 - 1612 . 1456334 10.2105/ajph.82.12.1607 PMC1694541

[ref029] Shi , L. , Samuels , M.E. , Pease , M. , Bailey , W.P. and Corley , E.H. ( 1999 ), “ Patient characteristics associated with hospitalizations for ambulatory care sensitive conditions in South Carolina ”, Southern Medical Journal , Vol. 92 No. 10 , pp. 989 - 998 . 10548172 10.1097/00007611-199910000-00009

[ref030] Starfield , B. , Shi , L. and Macinko , J. ( 2005 ), “ Contribution of primary care to health systems and health ”, The Milbank Quarterly , Vol. 83 , pp. 457 - 502 . 16202000 10.1111/j.1468-0009.2005.00409.x PMC2690145

[ref031] Statistics Canada ( 2013 ), Postal Code OM Conversion File (PCCF) , Reference Guide , Statistics Canada , Ottawa .

[ref032] von Bultzingslowen , I. , Eliasson , G. , Sarvimaki , A. , Mattsson , B. and Hjortdahl , P. ( 2006 ), “ Patients' views on interpersonal continuity in primary care: a sense of security based on four core foundations ”, Family Practice , Vol. 23 No. 2 , pp. 210 - 219 . 16361395 10.1093/fampra/cmi103

